# Blepharoconjunctivitis and Otolaryngological Disease Trends in the Context of Mask Wearing during the COVID-19 Pandemic

**DOI:** 10.3390/clinpract12040065

**Published:** 2022-08-11

**Authors:** Victoria A. Koshevarova, Zack K. Westenhaver, Mary Schmitz-Brown, Brian J. McKinnon, Kevin H. Merkley, Praveena K. Gupta

**Affiliations:** 1 School of Medicine, University of Texas Medical Branch, Galveston, TX 77555, USA; 2 Department of Ophthalmology, University of Texas Medical Branch, Galveston, TX 77555, USA; 3 Department of Otolaryngology, University of Texas Medical Branch, Galveston, TX 77555, USA

**Keywords:** COVID-19, mask, rhinitis, chalazion, blepharitis

## Abstract

(1) Purpose: In 2020, wearing of face masks was mandated in the United States in an effort to lessen transmission of the novel 2019 coronavirus disease (COVID-19) pandemic; however, long-term mask wearing may present with unintended side-effects in both ophthalmic and otolaryngologic clinical practice. This study aims to examine if mask wearing increased the incidence of primarily chalazion, blepharoconjunctivitis, and rhinitis occurrence during the mask-mandated COVID-19 pandemic period. (2) Methods: Medical records from tertiary academic center clinics were analyzed for incidence of ophthalmic and otolaryngologic diagnoses of interest (blepharoconjunctivitis- and rhinitis-related disorders). Data were collected from a pre-pandemic (March 2019–February 2020) and a mid-pandemic window (March 2020–February 2021) during which widespread mask mandates were implemented in Texas. Comparison was performed using a t-test analysis between incidence of chosen diagnoses during the described time periods. (3) Results: Incidence of ophthalmic disorders (primarily blepharoconjunctivitis and chalazion) in the pre-pandemic versus mid-pandemic windows did show a significant difference (*p*-value of 0.048). Similarly, comparison of otolaryngologic diagnoses (primarily rhinitis and related conditions) between the two time periods showed a significant difference (*p*-value of 0.044) as well. (4) Conclusion: Incidence of the chosen ophthalmic and otolaryngologic disorders did increase during periods of mask mandates. While these findings are preliminary, further studies are warranted to understand other factors that may have played a role in eye and nose pathology.

## 1. Introduction

In early to mid-2020, mask wearing was mandated throughout the United States in an effort to curb the spread of the novel 2019 coronavirus disease (COVID-19) brought on by the severe acute respiratory syndrome coronavirus 2 (SARS-CoV-2). On 3 April 2020, the Centers for Disease Control (CDC) officially recommended the wearing of face masks in public to lessen transmission, and in July 2020, wearing masks in public areas was mandated in the state of Texas [1]. Since then, attention has been drawn to the possibility that prolonged mask wearing may have an influence on ophthalmologic and otolaryngologic health. Three primary varieties of face masks were recommended for use by the CDC and the World Health Organization (WHO). These include cotton, surgical, and N-95 masks, all of which have shown efficacy in reducing transmission of COVID-19 via respiratory droplets [2]. A study on nasal irritation showed that the use of a filtering face piece could be associated with a new form of irritant rhinitis [3]. Conversely, there are reports that symptoms and incidence of allergic rhinitis seem to have decreased during the past year attributed to staying inside and wearing a mask [4,5]. Furthermore, studies of interest within the past year have shown that symptoms such as dry eye and chalazion have increased in incidence within clinics compared to before COVID-19, likely due to multifactorial causes [6,7,8,9]. Such causes are suspected to include mask wearing with consequential increased fomite transmission due to hand-to-face contact as well as changes in periorbital microenvironment due to redirected breathing from the mask. Increased screen time due to stay-at-home measures and sequelae of COVID-19 itself have also been noted to be probable causes to the increased incidence of these ophthalmic symptoms [6,7,8,9]. The purpose of this study is to retrospectively review patient charts to analyze whether wearing masks increases the risk of developing dry eyes, blepharitis, allergic conjunctivitis, chalazion, and hordeolum, allergic rhinitis, vasomotor rhinitis, chronic rhinitis, nasal irritation, and otalgia amongst patients at the University of Texas Medical Branch (UTMB) Eye and Ear, Nose, and Throat (ENT) clinics.

## 2. Materials and Methods

The study was approved by the institutional review board at the University of Texas Medical Branch and was conducted in accordance with the tenets of the Helsinki Declaration. Deidentified data was collected from academic institution clinics (UTMB Eye and Ears, Nose, and Throat [ENT] clinics, Galveston, TX, USA) in the form of a retrospective chart review. Medical records were analyzed for incidence of the diagnoses of interest (Table 1) for both a pre-pandemic window (March 2019–February 2020) and a mid-pandemic window (March 2020–February 2021) during which widespread mask mandates were implemented in Texas and UTMB clinics. Subjects were included based on age (18–100 years), presence of diagnosis (Table 1), and date seen in clinic. Patients who fell outside the specified age range, did not meet chosen diagnostic criteria or the diagnosis was not clearly documented, fell outside the study time windows, or presented with symptoms of interest but were associated with a comorbidity (e.g., Sjogren’s syndrome, post-operative follow-up, etc.) were excluded. Finally, data regarding COVID-19 trends was requested from the Galveston County Health District (GCHD) to evaluate for correlations with the incidence of the chosen diagnoses. Within figures, ophthalmic diagnoses (Table 1) of interest will henceforth be referred to as “chalazion and other disorders”, and ENT diagnoses (Table 1) will be termed “rhinitis and other disorders”.

## 3. Results

### 3.1. Ophthalmology Results

**Figure 1:**
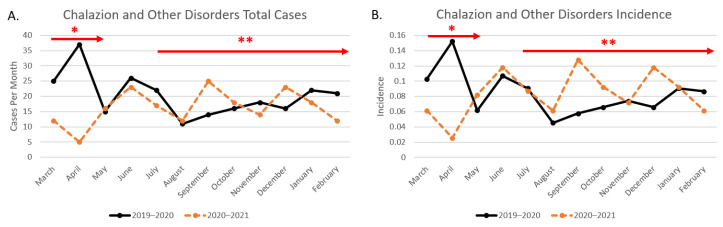
Total chalazion and other disorders cases including university lockdown and state mask mandate labels. Figure 1 shows the pre-pandemic versus mid-pandemic total cases per month (**A**) as well as the incidence (**B**). * Months during which the academic center was under lockdown (March 2019–early May 2019). ** Months during which Texas mask mandate was effective (July 2019–onward).

**Figure 2:**
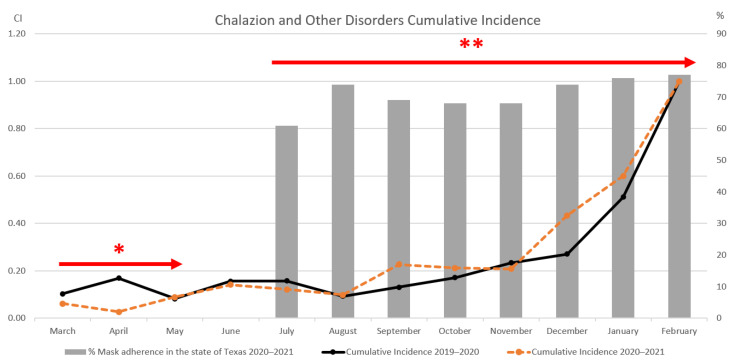
Cumulative chalazion and other disorder incidence with Texas mask adherence. During the UTMB lockdown period in early 2020 (arrow with one asterisk), the cumulative incidence from the 2019–2020 period was greater compared to the one from the 2020–2021 period. With the reopening of the academic center, the cumulative incidence from the 2020–2021 period started to slowly increase. Then from August 2020 to February 2021, during the state of Texas mask mandatory law (arrow with two asterisks), it started to drastically increase until overriding the 2019–2020 curve. As the percentage of mask adherence increases, the 2020–2021 cumulative incidence in the following month for chalazion and other disorders appeared to increase. * Months during which the academic center was under lockdown (March 2019–early May 2019). ** Months during which the Texas mask mandate was effective (July 2019–onward).

The total month-by-month cases and incidence from the pre-pandemic period (March 2019–February 2020) are compared to the chosen mid-pandemic period (March 2020–February 2021) in Figure 1. Red arrows indicating university clinic lockdowns and the beginning of the Texas mask mandate are also included for further data contextualization. The mid-pandemic year had fewer total cases (*n* = 195) compared to the pre-pandemic year (*n* = 243); hence, using incidence normalized as a percentage of total cases provides better insight as to the actual disease fluctuations. With the resolution of campus and clinic lockdown, the chalazion and other disorder incidence from the mid-pandemic period started to slowly increase between May and July, with spikes also noted in September and December 2021 (Figure 1). As the percentage of mask adherence increased (upward trend from November 2020 to February 2021 in Figure 2), the mid-pandemic cumulative incidence for chalazion and other disorders increased as well, and as the percentage of mask adherence decreased (downward trend from September–November 2020), the cumulative incidence for chalazion and other disorders decreased. A comparison of incidence using *t*-test analysis between the described time periods, assuming unequal variances, resulted in a *p*-value of 0.048. It should be noted that March was excluded from the analysis as the mask recommendations had not yet been implemented in Texas, and April was excluded due to statewide stay at home orders.

#### Otolaryngology Results

**Figure 3:**
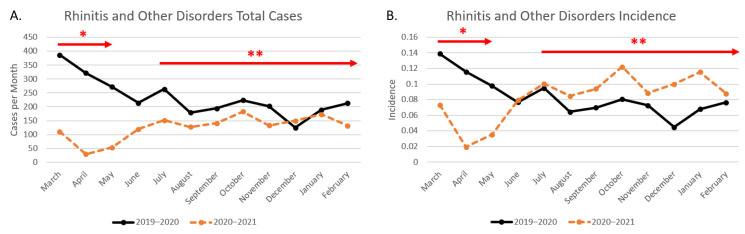
Total rhinitis and other otolaryngological disease cases including university lockdown and state mask mandate. This figure shows the pre-pandemic versus mid-pandemic total cases per month (**A**) as well as the incidence (**B**). * Months during which the academic center was under lockdown (March 2019–early May 2019). ** Months during which Texas mask mandate was effective (July 2019–onward).

**Figure 4:**
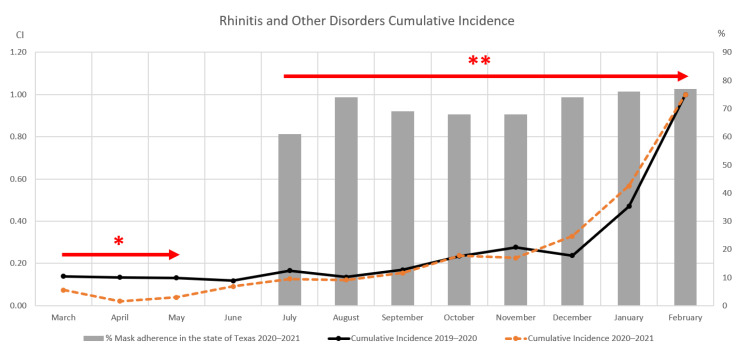
Cumulative rhinitis and other disorder incidence with Texas mask adherence. During the UTMB lockdown period in early 2020, the cumulative incidence from the 2019–2020 period was greater compared to the one from the 2020–2021 period. With the reopening of UTMB, the cumulative incidence from the 2020–2021 period started to slowly increase between May and October. Then from October 2020 to February 2021, during the state of Texas mask mandatory law, it started to drastically increase until overriding the 2019–2020 curve. As the percentage of mask adherence increases, the 2020–2021 cumulative incidence for rhinitis and other disorders appeared to increase. * Months during which the academic center was under lockdown (March 2019–early May 2019). ** Months during which the Texas mask mandate was effective (July 2019–onward).

The total month-by-month cases for allergic rhinitis and related disorders from the pre-pandemic period (March 2019–February 2020) is compared to the chosen mid-pandemic period (March 2020–February 2021) in Figure 3. Overall, the number of cases during the mid-pandemic period (*n* = 1501) was lower than that of the pre-pandemic window (*n* = 2785), and so using the incidence as a percentage of total cases provides better insight as to the actual disease fluctuation in this case as well. In Figure 4, cumulative incidence during the two periods is plotted against percentage of mask adherence beginning in July 2020. In the initial weeks after the lockdown period (arrow with one asterisk), Texas activity was slow as businesses reopened, and disease incidence appears to be higher in the 2019–2020 period. Between May and July, the number of cases increased from 50 to 153 at university clinics. Incidence in the mid-pandemic period continues to increase and eventually surpasses the pre-pandemic window around July 2020. When the two time periods are compared using *t*-test analysis assuming unequal variances, the cumulative incidence of otolaryngological disorders from 2019–2020 has a statistically significant difference from that of 2020–2021 with *p*-value of 0.044. Note that, like the ophthalmic statistical analysis described in Section 3.1 of the results, March and April were excluded from the comparison.

### 3.2. Galveston County Health District COVID-19 Data

**Figure 5:**
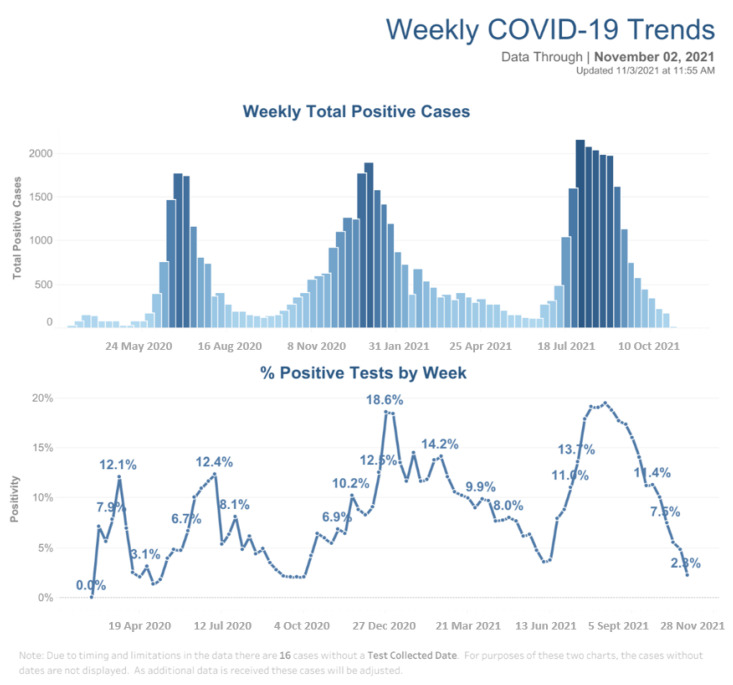
(Galveston County COVID-19 Weekly Positive Case Trends.) Overall COVID-19 cases in Galveston County from about March 2020 through November 2021.

Data from the Galveston County Health District regarding COVID-19 was obtained with permission and is shown in Figure 5 [10]. Peak total positive cases were seen in around July 2020, December 2020, and August 2021.

## 4. Discussion

The purpose of this study is to evaluate the incidence of ophthalmic and otolaryngologic diseases between a time period where masks were mandated in the state of Texas (March 2020–February 2021) and a control year (e.g., prior to the COVID-19 pandemic March 2019–February 2020). Based on the results, an increase in incidence was observed between ophthalmic and otolaryngologic diagnoses of interest in the 2020–2021 group versus the 2019–2020 group. Statistical analysis found significant differences in both groups (*p* < 0.05). Mask hygiene and mask fit are major components of mask efficacy [11]. Convenience coupled with the rising costs of masks may have led individuals to frequently re-use or recycle masks. Among non-health care professionals, mask quality and material varied. Cloth masks, while of moderate efficacy in preventing respiratory illness, are more cost effective as patients can recycle them but potentially compromise hygiene [3,11,12]. Without proper cleaning and overall hygiene regarding mask care, masks could cause nasal and eye irritation and consequently, related pathologies. This is one of the possible explanations for the trends noted in this study with respect to mask wearing. The ophthalmic results should be interpreted with some additional considerations. Although a significant trend relating masks and blepharoconjunctivitis-related disorders was noted within the patients, the smaller population size (*n* = 243 for the 2019–2020 group and *n* = 195 for the 2020–2021 group) may weaken the significance of the results. Other explanations have been cited as reasons for the increased incidence of these disorders during the pandemic. For instance, one proposition is that eye symptoms may be sequelae of COVID-19 (there is little data to support a similar proposition for rhinitis), which is why it was important to observe COVID-19 case trends alongside the chosen disease incidence [10,13]. As such, peaks in ocular cases showed some relationship (with a one to two month lag) with peaks in COVID-19 as shown in Figure 5. Eye symptoms may also be attributed to eye strain from extended time looking at a computer, especially due to the rise in work-from-home during the pandemic [9]. There are some considerations for the ENT disease incidence as well. The first is that air pollution and allergies were not included in the statistical analysis. Decrease in outdoor activities may have lowered the incidence of allergic rhinitis which would affect the 2020–2021 time-frame, especially during lockdown. However, despite the potential decrease in exposure to air pollution and allergies as people remained indoors during the quarantine, a relative increase in allergic rhinitis incidence was described by this study. Data gathered in this study is limited to Galveston and the surrounding area population, specifically those seen within the academic clinics. This can make it difficult to generalize the relationships observed in the study. Similarly, the data in this study may not represent that of Texas or United States. Mask adherence guidelines and the overall attitude towards masks was relaxed in 2021, likely due to multiple causes including social influence, politicization, and a reduced perceived threat of COVID-19 [14,15,16]. Because of this, the time period chosen to exhibit high mask adherence may not have been consistently representative. In addition to survey bias and regional biases towards masks, adherence may not be completely accurate and may be better viewed as a trend. The duration with which patients wear masks and choice of mask material are also difficult to predict and control for. This study did not concretely differentiate whether disease cases were new onset versus exacerbations of chronic processes, though some attempt at controlling for baseline trends was attempted via comparison to a “control” year. Lastly, as described in the ophthalmic discussion, eye and ENT disease in relation to COVID-19 trends was not closely analysed. Though Galveston county trends (Figure 5) were collected, quarantine precautions restricted in-person visits to patients who presented as COVID-19 negative, meaning eye and ENT diseases in COVID-19 positive patients may have gone undetected. This suggests that even with accurate COVID-19 trends, disease trend comparison may be unrepresentative, and more in-depth analysis would be outside the scope of this study.

## 5. Conclusions

This study addressed two important areas of consequence of mask wearing: eye and nose pathologies. However, because this study’s observations are based on one hospital-based analysis within a unique political and environmental context, clinicians should be careful in correlating similar findings at other clinics. It should also be noted that despite the trends gathered in this study, the benefits of wearing masks generally outweigh the risks in the context of COVID-19 spread, as evidenced by recent studies exemplifying the reduction of COVID-19 incidence (and other respiratory infection incidence) in populations of high mask adherence compared to those with low mask adherence [17,18,19]. Contradictory studies have noted multiple adverse effects amongst long-term mask wearers (with relevant ENT diseases including rhinitis and vocal cord disorders and other diseases falling under neurological, internal, dermatological, and psychiatric categories), which calls to attention the provider’s duty to weigh the overall risks and benefits of mask wearing to their patient in an unbiased and ethical way [20,21,22]. Nevertheless, like other similar studies on this topic [6,7], we recommend that instead of discouraging patients from wearing masks to reduce ocular or otolaryngological symptoms, providers should focus on educating patients on proper mask fit and hygiene. Future studies should investigate patients with history of eye and nose pathologies to highlight whether such findings are patient behavior linked (i.e., hygiene and mask wearing technique) or solely dependent on the presence of mask wearing. It is evident that more research about the long-term effects of masks is desirable to provide stronger guidelines on this topic.

## Figures and Tables

**Table 1:** List of ICD-10 codes analyzed.

Parent ICD-10 Code	Diagnosis
H00.0	Hordeolum
H00.1	Chalazion
H01.0	Blepharitis
H04.12	Dry Eye
H10	Conjunctivitis
H92.09	Otalgia
J30.0	Vasomotor Rhinitis
J30.9	Allergic Rhinitis
J31.0	Chronic Rhinitis
J34 Nasal	Irritation/Mucositis

## Data Availability

Data regarding COVID-19 cases in Galveston county, Texas, may be found at the following link: https://www.gchd.org/public-health-services/covid-19/covid-19-dashboard; https://www.gchd.org/public-health-services/covid-19/covid-19-dashboard (accessed on 2 November 2021).

## Abbreviations

The following abbreviations are used in this manuscript:

COVID-19	2019 coronavirus disease
SARS-COV-2	Severe acute respiratory syndrome coronavirus 2
CDC	Center for Disease Control
WHO	World Health Organization
UTMB	University of Texas Medical Branch
ENT	Ears, nose, and throat
GCHD	Galveston County Health District
MDPI	Multidisciplinary Digital Publishing Institute
DOAJ	Directory of open access journals
TLA	Three letter acronym
LD	Linear dichroism
